# 

**Published:** 1904-08

**Authors:** 


SUBSCRIPTION $1 00.

PUBLISHED MONTHLY

### ATLANTA JOURNAL-RECORD OF MEDICINE.

LIBRARY
INDEXED
S. G. O.

Successor to Atlanta Medical and Surgical Journal, Established 1855.

and Southern Medical Record, Established 1870.

Vol. VI. Atlanta, Ga., August, 1904. No. 5.

